# One-two punch mechanism of gene repression: a fresh perspective on gene regulation

**DOI:** 10.1007/s00294-017-0793-5

**Published:** 2017-12-07

**Authors:** Amy Tresenrider, Elçin Ünal

**Affiliations:** Department of Molecular and Cell Biology, Barker Hall, University of California, Berkeley, CA 94720 USA

**Keywords:** Meiosis, Gene regulation, Transcription, Translation, mRNA

## Abstract

Cellular differentiation depends on temporally controlled waves of gene activation and inactivation that ultimately transform one cell type into another. It is well established that transcription factor cascades coordinate the timely activation of gene expression clusters during development. In comparison, much less is understood about how gene repression events are coordinated with the transcription factor-driven waves of gene activation and how this repression is achieved at a mechanistic level. Using budding yeast as a model, we recently discovered a new gene regulatory event, whereby a central meiotic transcription factor induces the expression of an mRNA isoform to repress gene expression through an integrated transcriptional and translational mechanism. This new model could explain how gene activation and inactivation waves can be temporally coordinated. In this review, we discuss our findings and their potential implications.

## Introduction

The time and location of gene expression affect how organisms differentiate their cells into distinct lineages. While protein degradation and translational regulation affect the final level and localization of protein output, transcription factors are considered to be the dominant drivers of gene regulation throughout development. Well-known examples include the Hox genes and the Yamanaka factors. Hox gene expression patterns in the developing embryo dictate where along the anterior–posterior axis individual body parts will be in the adult organism (Krumlauf 1994). The Yamanaka factors—Oct4, Sox2, KLF4, c-Myc—are essential for maintaining stem cell pluripotency (Takahashi and Yamanaka 2006). Their expression is sufficient to reprogram differentiated cells into an undifferentiated state. Studies of these and other transcription factor families have provided invaluable insights into how gene activation can drive developmental programs. However, an equally important feature of cellular differentiation, that is gene repression, is often overlooked when considering transcription factor-driven gene expression programs. Consequently, the role of transcription factors in directly repressing gene expression remains understudied.

The teaching of central dogma states that one gene serves as a template for one type of mRNA. This mRNA in turn acts as a template for the production of a single protein product. It is widely accepted that this view is simplistic and does not capture the biological complexity of gene regulation. We now know that alternative splicing can produce hundreds of mRNA isoforms from a single gene. We know that non-coding RNAs make up a greater portion of the transcriptome than coding RNAs, and that these untranslated RNAs, such as micro-RNAs or long non-coding RNAs, play essential roles in organisms across eukaryotes (Brown et al. 1992; Lee et al. 1993; Mercer et al. 2009; Wightman et al. 1993). The mechanisms by which long non-coding RNAs affect gene regulation are varied. They can act in cis or trans, as antisense or intergenic transcripts, they can be either repressive or activating, be structural, alter chromatin states, or cause gene looping (Marchese et al. 2017; Sole et al. 2015; Wu et al. 2017). The unifying characteristic is that they are not translated. However, we are still very much attached to the notion that mRNA molecules (i.e., those that are capped, polyadenylated, and engaged with the ribosome) are translated. Their translation may be temporally delayed, as is the case for *CLB3* and *SSP2* in budding yeast meiosis, or their translation may only occur under specific circumstances as is the case for the upstream open reading frame (uORF) repressed *GCN4* and *ATF4* transcripts (Harding et al. 2000; Jin and Neiman 2016; Mueller and Hinnebusch 1986). In other words, translational repression is widely viewed as a switch-like mechanism, where translation of the ORF is repressed under certain conditions, but this repression can be bypassed under other conditions.

Recently, we uncovered a novel mechanism, where a developmental transcription factor induces the expression of an mRNA that serves a purely regulatory function. This mRNA is not translated into a functional protein due to the uORFs in its 5′ leader region (Chen et al. 2017). Instead, it serves to inactivate a gene through an integrated transcriptional and translational mechanism (Chen et al. 2017; Chia et al. 2017). This new insight challenges the assumption that mRNA molecules must produce the gene product encoded in their open reading frames and provides a fresh perspective on gene regulation.

## A 5′-extended mRNA represses kinetochore function

**Fig. 1:**
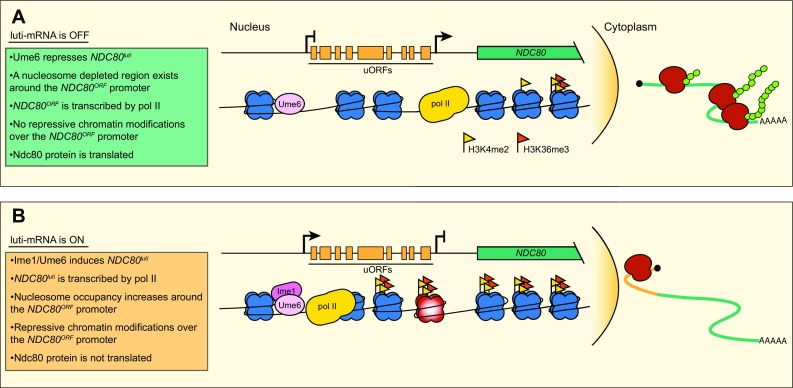
Schematic description of luti-mRNA gene regulation at the *NDC80* locus. In both panels, a depiction of the genomic *NDC80* locus is shown above a representation of the chromatin modifications and nucleosome positions at that locus. **a** *NDC80* ^*luti*^ is repressed by Ume6. The *NDC80* ^*ORF*^ promoter is depleted of nucleosomes. *NDC80* ^*ORF*^ is actively transcribed; it is exported to the cytoplasm and used by the ribosome to produce Ndc80 protein. **b** *NDC80* ^*luti*^ is expressed upon interaction of Ume6 with the meiotic transcription factor Ime1. Transcription of *NDC80* ^*luti*^ leads to an increase in both nucleosome occupancy and H3K4me2 and H3K36me3 chromatin modifications across the *NDC80* ^*ORF*^ promoter. Initiation of *NDC80* ^*ORF*^ is prevented. *NDC80* ^*luti*^ transcripts are exported to the cytoplasm and engaged by the ribosome. uORFs are translated, but Ndc80 protein is not translated from this mRNA

Our studies of kinetochore regulation during meiosis in budding yeast initially confirmed what we and others had seen previously: the essential kinetochore protein Ndc80 is downregulated during meiotic S-phase and prophase (Asakawa et al. 2005; Chen et al. 2017; Kim et al. 2013; Meyer et al. 2015; Miller et al. 2012; Sun et al. 2011). This assists in kinetochore remodeling which allows homologous chromosomes to be segregated in meiosis I. A deeper investigation into the mechanism by which the Ndc80 protein level decreases led us to discover an initially counterintuitive mechanism by which cells can downregulate gene expression in a cell (Fig. 1).

At the *NDC80* locus, a 5′-extended transcript isoform is expressed exclusively during meiosis. It is developmentally regulated by the master meiotic transcription factor Ime1 and its binding partner Ume6 (Bowdish et al. 1995; Chen et al. 2017; Park et al. 1992; Washburn and Esposito 2001). The extended transcript contains 9 uORFs in addition to the entire *NDC80* coding sequence. Using single molecule fluorescence in situ hybridization, we have shown that this transcript is exported from the nucleus. Its translation status, determined by ribosome profiling, confirms that the extended transcript is engaged with the ribosome (Brar et al. 2012; Chen et al. 2017; Miller et al. 2012). By all accounts, this RNA molecule would be considered an mRNA. Intriguingly, due to the uORFs, it cannot be translated. For this reason, we have termed this class of RNAs, luti-mRNAs, for long undecoded transcript isoform mRNAs and for which *NDC80* is the founding member. The undecoded *NDC80* isoform is referred to as *NDC80* ^*luti*^, while the canonical transcript, which is translated into Ndc80 protein, is referred to as *NDC80* ^*ORF*^.

In addition to being translationally repressed, *NDC80* ^*luti*^ expression blocks transcription initiation at the *NDC80* ^*ORF*^ promoter. When Ime1 induces *NDC80* ^*luti*^, RNA polymerase II (pol II) complex is recruited to the distal promoter and transcription elongation occurs across the proximal *NDC80* ^*ORF*^promoter. This causes an increase in repressive histone modifications, namely H3 lysine 4 dimethylation (H3K4me2) and H3 lysine 36 trimethylation (H3K36me3), over what had previously been an active promoter (Chia et al. 2017). Both marks are co-transcriptionally deposited in a pol II-dependent manner and are involved in repressing cryptic transcription initiation within gene bodies (Carrozza et al. 2005; Keogh et al. 2005; Kim and Buratowski 2009; Li et al. 2003; Ng et al. 2003; Xiao et al. 2003). Their deposition at the *NDC80* ^*ORF*^promoter, however, correlates with the repression of a previously active promoter. Furthermore, an increase in nucleosome occupancy upon *NDC80* ^*luti*^ expression is dependent on the conserved chromatin associated proteins: Set3, a member of the Set3C histone deacetylase complex, and Set2, an H3K36me3 methyltransferase (Pijnappel et al. 2001; Strahl et al. 2002). When deposited over the canonical *NDC80* promoter, H3K4me2 and H3K36me3 together increase nucleosome occupancy, which in turn inhibits transcription initiation at the *NDC80* ^*ORF*^promoter (Chia et al. 2017).

When we consider both the transcriptional and translational methods utilized by the cell to affect Ndc80 protein levels together, a fuller picture of gene regulation at this locus begins to emerge. An mRNA transcript, from which the ribosome does not translate full-length protein, still serves a key function. The act of its transcription leads to the recruitment of chromatin modifying enzymes—thought to prevent spurious initiation within gene bodies. In the case of *NDC80*, initiation of transcription upstream of active promoters serves as a cis-mediated mechanism to prevent transcription of downstream mRNA isoforms during a developmental program. This unexpected one-two punch of both transcriptional and translational repression in such a non-canonical manner calls for further investigation for other instances by which the integration of multiple steps in gene regulation determines the final gene output. In addition, we must not forget to consider the role of protein stability in all gene expression studies as evidence suggests that Ndc80 is actively degraded in meiotic prophase (Jingxun Chen, personal communication).

Later in meiosis, kinetochores must be active in order to segregate chromosomes. This occurs through the re-expression of Ndc80 protein. Instead of changing the translational status of *NDC80* ^*luti*^ as in the case of *GCN4*, the cells evolved a different solution to produce Ndc80 protein. This strategy relies on a switch in promoter usage, from distal to proximal, which is dictated by at least two events: The first is the reduction in Ime1 levels as cells progress through meiosis (Brar et al. 2012). This leads to reduced expression of *NDC80* ^*luti*^, which likely dampens luti-mRNA-mediated repression of *NDC80* ^*ORF*^. Second, and more importantly, the increased activity of Ndt80, a transcription factor known to be responsible for the subsequent major wave of gene expression, induces the protein coding *NDC80* mRNA isoform, *NDC80* ^*ORF*^ (Chen et al. 2017; Chu and Herskowitz 1998; Xu et al. 1995). As a result, Ndc80 protein is expressed, permitting re-activation of kinetochores to segregate meiotic chromosomes. This finding further highlights how a developmental switch in promoter usage can cause coordinated expression of two disparate mRNA isoforms to achieve precise temporal control of protein translation during cellular differentiation.

## luti-mRNAs in additional contexts

A luti-mRNA as we have defined it has three essential features: (1) it has a 5′-extended leader sequence, (2) it is not decoded by the ribosome and, therefore, does not produce a functional full-length protein, and (3) it is regulated by condition-specific transcription factors. Additionally, for a luti-mRNA to repress gene expression, it must prevent transcription initiation from the downstream gene promoter. This final feature is not necessary for a transcript to be called a luti-mRNA, but it is necessary for it to have the strongest repressive effect on gene expression. Considering that alternative start site usage (Aanes et al. 2013; Batut et al. 2013; Kimura et al. 2006), translation of repressive uORFs (Calvo et al. 2009; Chew et al. 2016; Johnstone et al. 2016), conditional regulation of gene expression by transcription factors, and inhibition of transcription initiation at sites of overlapping transcription have all been individually observed in organisms from yeast to humans (Corbin and Maniatis 1989; Eissenberg and Shilatifard 2010; Shearwin et al. 2005; Wagner and Carpenter 2012), we speculate that the luti-mRNA mechanism of gene repression is widespread.

In budding yeast, hundreds of transcripts with 5′-extended leaders are expressed in meiosis; the majority of which also contain translated AUG initiated uORFs, as observed by ribosome profiling (Brar et al. 2012). On an individual gene basis, *BOI1*, which is involved in polarized growth, has an extended meiosis-specific transcript that, similar to *NDC80* ^*luti*^, is repressed by the mitotic repressor of meiotic genes Ume6 (Liu et al. 2015). In another case, it was demonstrated that the origin of recognition gene *ORC1* has an extended transcript that is regulated by the mid-meiotic transcription factor Ndt80 (Xie et al. 2016). We predict that other extended meiotic transcript isoforms are regulated by these two master transcription factors and that upon further investigation many will prove to be translationally repressed luti-mRNAs.

Outside of meiosis, 5′-extended transcripts appear when budding yeast cells are shifted between carbon sources such as from dextrose to galactose. This appears to rely on the same chromatin-associated proteins Set2 and Set3 that are necessary for *NDC80* ^*ORF*^ transcriptional repression (Kim et al. 2016, 2012). The extent to which the carbon source-dependent extended transcripts are translationally inhibited is still unknown. Implementation of techniques such as ribosome profiling may uncover a mechanism of translational repression in addition to transcriptional repression just as in the case of *NDC80*.

In addition, a recent paper described a phenomenon where, upon zinc depletion, budding yeast cells induce the expression of 5′-extended transcript isoform at the *RTC4* gene locus. This is mediated by the transcription factor Zap1 and produces a transcript that is not translated into Rtc4 protein (Taggart et al. 2017). Besides *NDC80*, this is the only frequently referred to as theother case of what could be referred to as a bona fide luti-mRNA, because both the transcriptional regulation and the translational status of the extended isoform were assessed. Their observation of a decrease in the coding *RTC4* transcript and Rtc4 protein upon zinc starvation points towards a yet-to-be-determined mechanism by which the extended *RTC4* transcript represses downstream transcription initiation. Increases in H3K4me2 and H3K36me3 across the promoter of the shorter transcript may very well prove to be involved again.

These three examples, where luti-mRNAs appear to be expressed, all represent rapidly changing conditions where gene expression is highly dynamic. Therefore, the new cases of luti-mRNAs are likely to be observed in analogous contexts such as during differentiation or in response to environmental stress. Yeast meiosis, with its dynamic gene expression pattern and tractability, has enabled us to dissect each step at which a luti-mRNA affects gene regulation. We can now use this information as a roadmap to explore luti-mRNAs in more complex genomes.

Approximately, 50% of mouse, > 40% of drosophila, and 30–50% of human genes have alternative start site usage during development (Batut et al. 2013; Kimura et al. 2006). In those same organisms, uORF translation is prevalent with an estimated 50% of human genes harboring translated uORFs (Calvo et al. 2009; Chew et al. 2016; Dunn et al. 2013; Johnstone et al. 2016). Transcription-coupled chromatin modifications are also highly conserved across evolution (Eissenberg and Shilatifard 2010; Wagner and Carpenter 2012). Therefore, it does not seem far-fetched to propose that the form of regulation controlling the *NDC80* locus in budding yeast meiosis could be responsible for fine-tuning gene expression in other organisms.

One intriguing example comes from the study of gene regulation during neuronal differentiation of human embryonic stem cells. The translational efficiency (TE) of hundreds of genes varies during this developmental process. It was recently shown that the genes with variable TE during differentiation also experience changes in ribosome occupancy over their 5′ leaders. Furthermore, the authors concluded that the changes in TE are dominated not by changes in the ribosome composition but by transcript isoform usage (Blair 2017). Could these be luti-mRNAs? Investigation of the transcriptional regulation controlling isoform usage would shed more light on the role that luti-mRNAs may play in neuronal differentiation.

## Why luti-mRNAs?

Upon first glance, this mode of gene regulation appears counterintuitive. Why would a cell utilize transcription activators to inhibit gene expression? Why would a cell transcribe mRNAs that cannot produce protein? Why would a cell repress gene promoters by distal transcription of mRNA isoforms? Although the “why” questions are extremely difficult to answer in biology, we will describe our perspective on the logic of this gene repression mechanism.

Over evolutionary time, it is thought that cis-regulatory mechanisms are the dominant drivers of changes in gene expression (Carroll 2008; Stern and Orgogozo 2008; Wittkopp and Kalay 2011). Most developmental processes use a small number of transcription factors to turn on hundreds or even thousands of targets. If the transcription factor was to change in function, it would affect the levels of all of its targets, likely to the detriment of the organism. However, small changes to the regulatory regions of target genes allow for the tuning of gene expression (Carroll 2008). We propose that this is how luti-mRNA regulation arose. Small changes in the regulatory sequence that co-opted existing transcription factors to produce 5′ extended transcript isoforms enabled these genes to now be controlled by the luti-mRNA mechanism. These isoforms most likely already had uORFs, as short open reading frames are pervasive outside of coding regions (Chew et al. 2016). Thus, mutations in the regulatory regions over time may have evolved to allow transcription factors to behave as both activators and repressors. Through this pathway, transcription factors would gain a wider range of regulatory potential without having to evolve novel trans-acting factors.

Having the same transcription factor tuning gene expression both up and down could also provide regulatory advantages in development. As stated above, a few transcription factors usually regulate a large number of targets. In the case of budding yeast meiosis, the relay between two transcription factors, Ime1 and Ndt80, controls the landmark events in meiotic differentiation (Chu and Herskowitz 1998; van Werven and Amon 2011; Xu et al. 1995). In contrast, a situation in which a transcriptional activator  must be coordinated with the activity of a transcriptional repressor to produce the same gene expression output would require more “parts” and more co-regulation than what we have observed. Furthermore, inhibiting the expression of genes, whose functions are no longer required, or can even be detrimental for a given developmental stage, is as important as activating gene expression. Therefore, luti-mRNAs provide a clever solution to temporally coordinate gene repression with the transcription-factor driven waves of gene activation.

The prototype view of uORF-mediated translational regulation comes from the elegant studies of *GCN4*, the transcriptional activator of amino acid biosynthetic genes (Mueller and Hinnebusch 1986). *GCN4* expression is controlled by a switch-like mechanism where the uORFs repress translation of the *GCN4* mRNA in nutrient-rich conditions, but this translational repression is relieved upon nutrient starvation (Mueller and Hinnebusch 1986). In other words, whether or not a cell expresses Gcn4protein under different conditions is primarily determined by the translational status of a single *GCN4* mRNA isoform. In the case of luti-mRNAs, the uORF-mediated translational repression renders the luti-mRNA permanently non-coding. Because of the perpetual nature of this translational repression, whether or not a cell expresses protein under different conditions is instead determined by a switch in promoter usage. The promoter choice for *luti versus coding* mRNA ultimately determines whether a gene is turned on or turned off by a given transcription factor. In addition to tuning the translational capacity of a given mRNA, we propose that the function of some uORFs is to completely shutdown translation of regulatory luti-mRNAs.

In luti-mRNA-mediated gene repression, the luti-mRNA contains the entire ORF of a protein in addition to its extended 5′-leader. Why might cells not just produce short, prematurely terminated transcripts? In the case of *NDC80* ^*luti*^, premature termination of the transcript abrogates its repressive activity (Chen et al. 2017). It is possible that, for genes prone to repression by luti-mRNAs, transcription termination before the proximal promoter would not recruit sufficient levels of repressive chromatin modifications and would not affect nucleosome position. If true, then the transcript should not be terminated before the proximal promoter, but it should also not be terminated within the gene, so as to prevent disruption of the ORF in the coding mRNA isoforms. As a result,  repressive luti-mRNAs contain entire ORFs. However, there are cases of non-coding intergenic RNAs in budding yeast, such as *SER3* and *IRT1* where sense transcription near the downstream promoter prevents initiation (Martens et al. 2004; van Werven et al. 2012). It is possible that a prematurely terminated transcript can give rise to repression, but only if the terminator is directly adjacent to the proximal promoter. The pervasiveness of this configuration remains to be examined.

Finally, luti-mRNA-mediated gene repression is highly dynamic and tunable. While such plasticity may be especially critical during cell fate transitions to enable rapid adaptation to internal as well as external changes, it could also impact gene expression programs in a broader biological context such as during signaling and metabolic pathways. The repressive chromatin marks, which are established at proximal gene promoters as a result of luti-mRNA transcription, can be rapidly reversed. In the case of *NDC80* ^*luti*^, when its expression is halted, the chromatin modifications are removed within 15 min, followed by the return of *NDC80* ^*ORF*^ and Ndc80 protein expression (Chia et al. 2017). This, along with recent studies of chromatin remodelers and histone demethylases, prompts us to revisit the question of how stable chromatin modifications are (Perino and Veenstra 2016). However, it is worth noting that in organisms with DNA methylation, luti-mRNA regulation could serve to recruit H3K36me3-dependent DNA methyltransferases, such as DNMT3B, to permanently silence gene expression (Jeziorska et al. 2017; Morselli et al. 2015; Neri et al. 2017).

## A future for luti-mRNA identification

To identify luti-mRNAs, both transcription and translation must be studied hand in hand. RNA-seq has long been a staple of gene expression studies, but as ribosome profiling has demonstrated, studying RNA abundance alone does not reflect protein abundance in the cell (Ingolia et al. 2009). Codon optimality, 3′ UTR features, and 5′ UTR features are all expected to affect how well a transcript is translated (Barrett et al. 2012; Pop et al. 2014). Our investigation has conclusively demonstrated that uORFs in the 5′-leader of *NDC80* ^*luti*^ (frequently referred to as the 5′ UTR) do affect how well the transcript is translated, but that is not the only reason why translation decreases when *NDC80* ^*luti*^is expressed. The repression of *NDC80* ^*ORF *^by increased repressive chromatin modifications and nucleosome occupancy decreases the abundance of the isoform necessary for productive translation of Ndc80 protein (Chia et al. 2017). Without considering both the transcriptional and translational effects that a single transcript isoform could have on gene regulation, our mechanism would have been overlooked.

The first hurdle in identifying luti-mRNAs in other organisms will be identifying potential luti-mRNA transcript isoforms with high confidence. Recent advances in long read sequencing technologies have opened the door for transcript isoform identification. Both PacBio and Nanopore sequencing technologies can sequence entire mRNA transcripts with a single read (Au et al. 2013; Bolisetty et al. 2015). This circumvents the need to fragment the nucleic acid before performing RNA-seq. Instead of relying on maximum likelihood modeled transcript identification, these “3rd generation” sequencing platforms provide direct evidence for transcript isoform expression. Technologies such as these will be invaluable in identifying true full-length luti-mRNAs, which contain the entire ORF of a gene, from truncated transcripts that only partially overlap with a gene’s ORF (Table 1). Once putative luti-mRNA isoforms are identified, techniques such as ribosome profiling can be utilized to call which transcripts have translated uORFs (Ingolia et al. 2009) (Table 1). The translational status of the luti-mRNAs can be further dissected by Transcript Isoforms in Polysomes sequencing (TrIP-seq) (Floor and Doudna 2016) (Table 1).

**Table 1:** Methods to identify luti-mRNAs

luti-mRNA characteristic	Method for genome-wide identification
1. Extended 5′ leader	Nanopore or PacBio sequencing
2. Temporally regulated expression	Transcription factor ChIP-seq or ChIP-exo
3. Poor translational efficiency	TrIP-seq, ribosome profiling
4. Repression of a downstream ORF	ChIP-seq, MNase-seq, Nanopore or PacBio sequencing in mutant cells

The final aspect of luti-mRNA-mediated repression that must be further characterized is its ability to affect protein output. This will depend on how stable the protein is. Classical methods such as immunoblotting and reporter assays can reveal changes in protein expression. However, recent advances in quantitative mass spectrometry will allow this third layer of gene regulation to be investigated in the context of luti-mRNAs on a proteome-wide scale. Without considering effects on protein levels, the impact of luti-mRNA regulation cannot be fully understood. This is yet another reason why we should perform studies that integrate analyses at each step of gene expression. Without such comprehensive studies, we have only a limited view of gene regulation and can merely speculate as to what other mechanisms cells may be employing to ensure that gene expression is accurate and reproducible throughout development.
